# A heatmap for expected cumulative live birth rate in preimplantation genetic testing for monogenic disorders and chromosomal structural rearrangements

**DOI:** 10.1007/s10815-024-03141-6

**Published:** 2024-05-16

**Authors:** Annelore Van Der Kelen, Kathelijn Keymolen, Wilfried Cools, Anick De Vos, Laura Pölsler, Michel De Vos, Christophe Blockeel, Elia Fernandez-Gallardo, Martine De Rycke, Veerle Berckmoes, Pieter Verdyck, Frederik Jan Hes, Willem Verpoest

**Affiliations:** 1 Clinical Sciences, Research Group Genetics, Reproduction and Development, Centre for Medical Genetics, Vrije Universiteit Brussel (VUB), Universitair Ziekenhuis Brussel (UZ Brussel), Laarbeeklaan 101, 1090 Brussels, Belgium; 2 Support for Quantitative and Qualitative Research, Vrije Universiteit Brussel (VUB), Laarbeeklaan 103, 1090 Brussels, Belgium; 3 Clinical Sciences, Research Group Genetics, Reproduction and Development, Brussels IVF Centre for Reproductive Medicine, Vrije Universiteit Brussel (VUB), Universitair Ziekenhuis Brussel (UZ Brussel), Laarbeeklaan 101, 1090 Brussels, Belgium; 4 Department of Reproductive Medicine, Utrecht University Medical Centre (UMCU), Utrecht University, Heidelberglaan 100, 3584 CX Utrecht, The Netherlands

**Keywords:** AMH, Cumulative live birth rate (CLBR), Female age, Preimplantation genetic testing for monogenic disorder (PGT-M), Preimplantation genetic testing for chromosomal structural rearrangements (PGT-SR)

## Abstract

**Purpose:**

Our objective is to predict the cumulative live birth rate (CLBR) and identify the specific subset within the population undergoing preimplantation genetic testing for monogenic disorders (PGT-M) and chromosomal structural rearrangements (PGT-SR) which is likely to exhibit a diminished expected CLBR based on various patient demographics.

**Methods:**

We performed a single-centre retrospective cohort study including 1522 women undergoing 3130 PGT cycles at a referral centre for PGT. A logistic regression analysis was performed to predict the CLBR per ovarian stimulation in women undergoing PGT-M by polymerase chain reaction (PCR) or single-nucleotide polymorphism (SNP) array, and in women undergoing PGT-SR by SNP array, array comparative genomic hybridization (CGH) or next-generation sequencing (NGS).

**Results:**

The mean age of women was 32.6 years, with a mean AMH of 2.75 µg/L. Female age and AMH significantly affected the expected CLBR irrespective of the inheritance mode or PGT technology. An expected CLBR < 10% was reached above the age of 42 years and AMH ≤ 1.25 µg/L. We found no significant difference in outcome per ovarian stimulation between the different PGT technologies, i.e. PCR, SNP array, array CGH and NGS. Whereas per embryo transfer, we noticed a significantly higher probability of live birth when SNP array, array CGH and NGS were used as compared to PCR.

**Conclusion:**

In a PGT-setting, couples with an unfavourable female age and AMH should be informed of the prognosis to allow other reproductive choices. The heatmap produced in this study can be used as a visual tool for PGT couples.

**Supplementary Information:**

The online version contains supplementary material available at 10.1007/s10815-024-03141-6.

## Introduction

For many decades, couples at risk of transmitting a genetic condition to their offspring could only opt for prenatal testing. In the early nineties, preimplantation genetic testing (PGT) was developed as an alternative to prenatal testing and subsequent termination of pregnancy [1]. Since its introduction, indications have gradually expanded and today PGT can be offered for monogenic disorders, chromosomal structural rearrangements and aneuploidies simultaneously [2].

Couples undergoing PGT for monogenic disorders are usually fertile. Hence, at any given age a relatively normal to high ovarian response, high fertilization rates and normal embryo cleavage rates are expected for couples undergoing PGT for genetic indications that are not associated with reduced ovarian reserve. On the other hand, a proportion of the obtained embryos will be affected by the disorder in question, and a small proportion of the embryos will remain without a genetic test result and may not be transferred. The percentage of embryos not suitable for embryo transfer as a result of PGT for monogenic disorders (PGT-M) varies between 25 and 87.5%, depending on the indication for PGT [3]. Additional aneuploidy screening of embryos using the more recent genomic assays further increases the number of unsuitable embryos. Consequently, the population undergoing PGT actually shows a lower cumulative live birth rate (CLBR), defined as the likelihood of achieving at least one live birth per ovarian stimulation, compared to a conventional IVF population [4–6].

Nevertheless, in view of the prior absence of fertility problems, couples undergoing PGT often have high expectations. It is therefore important to provide these couples with accurate information in order to attenuate expectations. Couples for whom the expected CLBR is too low should be informed of the prognosis and counselled regarding other reproductive options, such as spontaneous conception with prenatal testing, gamete donation, adoption, foster care or remaining childless.

In 2021, our group reported the CLBR for PGT-M using polymerase chain reaction (PCR) technology. We showed that the CLBR was significantly influenced by female age, the number of previous medically assisted reproduction (MAR) cycles, the number of oocytes and the dose of gonadotropins for ovarian stimulation [6]. Recently, anti-Mullerian hormone (AMH) gained widespread use as a biomarker of ovarian reserve and response after ovarian stimulation in conventional IVF [7]. In addition, PCR technology is nowadays increasingly being replaced by genome-wide approaches, such as single-nucleotide polymorphism (SNP) array, array comparative genomic hybridization (CGH) and next-generation sequencing (NGS) technologies [8]. A tool to calculate the expected CLBR for PGT cycles using these latter technologies has not been developed to date. Therefore, we assessed the expected CLBR for women undergoing PGT based on PCR, SNP array, array CGH or NGS technologies, and we propose an expected CLBR below 10%, 5% and 1% as thresholds for a poor, a very poor or a futile prognosis, respectively [6].

The principal aim of the present study was the identification of clinical parameters, including AMH, to predict the cumulative live birth in women undergoing PGT-M or PGT-SR. A secondary aim was to compare the expected CLBR for different PGT technology groups, i.e. PCR, SNP array, array CGH or NGS.

## Materials and methods

### Study population

We performed a single-centre retrospective cohort study, covering cycles of women undergoing a PGT-M or PGT-SR treatment between 01/01/2015 and 31/08/2020. All females were aged between 18 and 45 years old at the time of oocyte retrieval (OR). The data obtained included female age, AMH, BMI, indication for PGT, inheritance mode of the disorder, number of previous unsuccessful PGT cycles, year of treatment and PGT technology (PCR, SNP array, array CGH or NGS). The outcome of a live birth per ovarian stimulation, including both transfer of fresh as well as frozen embryos, was extracted from the medical files (i.e. CLBR). This definition also includes those cycles wherein ovarian stimulation did not proceed to oocyte retrieval due to various reasons such as limited ovarian response, patient dropout and medical reasons. We also extracted the live birth rate (LBR), defined as the rate of a live birth per embryo transfer. Only outcomes available before June 2021 were included. Exclusion criteria consisted of the following: cycles making use of in vitro maturation (IVM), fine-needle aspiration (FNA) or testicular biopsy (TESE) for sperm retrieval, oocyte donation, modified natural cycles (MNC), warmed oocyte embryo transfers (WOET), PGT for aneuploidies (PGT-A), as well as PGT-M for indications that are known to be associated with reduced ovarian response (such as *FMR1* premutations) and PGT-SR for X- and/or Y-chromosome translocations. Subsequently, all included cycles were divided into four categories based on the PGT technology used: PCR, SNP array, array CGH or NGS.

### ICSI treatment and genetic testing

All women underwent intra-cytoplasmic sperm injection (ICSI) followed by embryo biopsy, genetic testing and transfer of unaffected embryos. Prior to ICSI treatment, a preclinical work-up was performed for all cycles using PCR and SNP array technology. During this work-up, DNA samples from the couple and relevant family members were used for the identification of genetic markers (i.e. small tandem repeats (STR) or SNPs) close to the region of interest (i.e. haplotyping). High-risk haplotypes are those shared by family members who carry the familial (likely) pathogenic variant, as opposed to wild-type or low-risk haplotypes which are present in unaffected family members. The clinical PGT test can be direct, which involves specifically evaluating the (likely) pathogenic variant and associated genetic markers, or indirect, which involves testing based solely on haplotyping [2, 9]. No preclinical work-up was needed to determine haplotypes for cycles using array CGH and NGS technologies. Conventional stimulation protocols with GnRH agonist or antagonist were used as ovarian stimulants [10]. In the case of PGT with PCR, a single-cell biopsy was performed on day 3 embryos after opening the zona pellucida using two or three laser pulses of 5–7 ms from a non-contact 1.48-mm diode laser system (Octax Laser Shot, Octax Microscience GmbH, Germany, using Octax Eye Ware software or Saturn 5, Research Instruments, UK) [11], followed by a fresh or frozen embryo transfer on day 5 or 6 of embryo development [12]. In the case of PGT with SNP array, array CGH or NGS, a trophectoderm biopsy at day 5 or 6 was performed after opening of the zona pellucida using two or three laser pulses, after which five to seven cells were aspirated and laser-excised or obtained by mechanical pulling [13]. All embryos were cryopreserved whilst genetic test results were pending. This was followed by a frozen embryo transfer in a deferred cycle [12]. Embryos were only suitable for transfer when several criteria were met. First, they were unaffected by the genetic disorder in question (PGT-M) or were normal or balanced (PGT-SR). Second, they were normal at the locus of interest (maternal and paternal haplotype present for PCR) or euploid when using a genome wide technology (SNP array, aCGH or NGS). Third, they met morphological criteria as described by Gardner [14]. When supernumerary embryos were available, they could potentially be transferred in a following menstrual cycle after vitrification [15]. The transfer of a thawed embryo was conducted either in a natural cycle or in a hormone replacement therapy cycle.

### Statistics

Calculation of the mean and standard deviation (SD) were applied for continuous outcomes, whereas (relative) frequency was applied for dichotomous outcomes. Because our dataset consisted of multiple observations per woman, a random-intercept generalized linear mixed model was considered, with logit link for the prediction of the CLBR per ovarian stimulation. Where the random intercept variance was estimated as zero, suggesting little heterogeneity among patients, the model is simplified to an ordinary logistic regression. The following predictors were considered stepwise using the Akaike information criterion (AIC): female age, AMH, BMI, inheritance mode, number of previous unsuccessful PGT cycles, year of treatment, PGT technology, day of biopsy (day 3 vs. day 5) and the type of transfer (fresh vs. frozen). For continuous predictors that remained in the model, a polynomial extension was considered. For the prediction of the LBR per transfer, a similar model was applied with the following parameters: female age, BMI, inheritance mode, the number of previous unsuccessful PGT cycles, year of treatment, PGT technology, day of biopsy (day 3 vs. day 5) and the type of transfer (fresh vs. frozen). Post hoc analysis, using Tukey pairwise comparison, was used for categorical predictors that remained in the model. The model was used to create a prognosis based on female age and AMH, and was visualized with heatmaps which may find application for counselling purposes. In addition, a non-inferiority test allowing a margin of 5% on the actual estimate was used to compare PCR with SNP array technology.

### Ethics

All data underlying this study were appropriately safeguarded and encrypted in accordance with the General Data Protection Regulation (GDPR). The Institutional Review Board of the Universitair Ziekenhuis Brussel gave their approval for the start of the study (B.U.N.: 1,432,021,000,498) as well as for the continuation, based on annual reports.

## Results

### Baseline characteristics

The overall analysis encompassed a total of 3037 cycles in 1490 women. The data included 749 fresh embryo transfers in 484 women and 2065 vitrified-warmed embryo transfers in 1000 women. The characteristics of the study population are summarized in Table 1. Between 2015 and 2020, most cycles were performed using PCR technology (60.1%), followed by SNP array (23.3%), array CGH (9.6%) and NGS (7.0%). The study population had a mean female age of 32.6 years (SD 4.4), a mean BMI of 24.3 kg/m^2^ (SD 4.4), a mean AMH of 2.75 µg/L (SD 2. 60) and an average of 0.98 previously unsuccessful completed PGT cycles (SD 1.40). The included cycles used a mean of 2313.28 IU (SD 857.36) gonadotrophins for ovarian stimulation and on average 11.9 oocytes (SD 7.5) could be retrieved per cycle. The mean number of embryos biopsied was 4.9 (SD 4.1), and a mean of 1.6 (SD 1.8) embryos were suitable for transfer, with 60% of cycles having at least one embryo transfer. The expected CLBR per ovarian stimulation for the whole study population reached 34% and was similar across the different subgroups. However, the expected LBR per transfer was higher in the SNP array (49%), array CGH (45%) and NGS (51%) subgroups compared to the PCR subgroup (37%).

**Table 1:** Characteristics and outcomes of the total study population and of four subgroups that underwent different genomic analyses (PCR, SNP array, array CGH, and NGS). The number of cases, the female and cycle characteristics, as well as the pregnancy outcome are presented for the total study population as well as for the four subgroups. Number (n.), year (y.), international units (IU), body mass index (BMI), anti-Mullerian hormone (AMH), preimplantation genetic testing (PGT), cumulative live birth rate (CLBR), live birth rate (LBR), embryo transfer (ET)

	PCR	SNP array	Array CGH	NGS	Total
Number of cases					
Cycles (*n*.)	60.1% (*n* = 1824)	23.3% (*n* = 707)	9.6% (*n* = 293)	7.0% (*n* = 213)	100% (*n* = 3037)
Embryo transfers (*n*.)	69.2% (*n* = 1931)	18.0% (*n* = 484)	5.0% (*n* = 221)	7.8% (*n* = 132)	100% (*n* = 2814)
Women (*n*.)	57.8% (*n* = 861)	26.0% (*n* = 388)	10.9% (*n* = 162)	8.8% (*n* = 131)	100% (*n* = 1490)
Female characteristics					
Female age (y.)	32.16 (± 4.23)	33.32 (± 4.68)	33.38 (± 4.27)	32.45 (± 4.45)	32.57 (± 4.39)
BMI (kg/m^2^)	24.13 (± 4.45)	24.49 (± 4.38)	24.17 (± 4.10)	24.59 (± 4.84)	24.25 (± 4.43)
AMH (µg/L)	2.74 (± 2.61)	2.65 (± 2.24)	3.00 (± 3.57)	2.84 (± 2.02)	2.75 (± 2.60)
Cycle characteristics					
Previous PGT cycles (*n*.)	1.05 (± 1.40)	0.84 (± 1.31)	1.03 (± 1.77)	0.84 (± 1.08)	0.98 (± 1.40)
Stimulation units (IU)	2284.50 (± 845.59)	2332.78 (± 885.04)	2368.23 (± 846.15)	2419.50 (± 871.76)	2313.28 (± 857.36)
Retrieved oocytes (*n*.)	11.72 (± 7.20)	12.38 (± 8.07)	11.23 (± 7.81)	12.32 (± 7.04)	11.87 (± 7.46)
Embryos biopsied (*n*.)	5.97 (± 4.38)	3.27 (± 3.09)	3.27 (± 3.03)	2.67 (± 2.59)	4.86 (± 4.12)
Embryos for transfer (*n*.)	1.69 (± 1.70)	1.45 (± 1.90)	1.53 (± 1.82)	1.19 (± 1.47)	1.59 (± 1.75)
Cycles with transfer (*n*.)	68% (*n* = 1243)	49% (*n* = 344)	48% (*n* = 142)	46% (*n* = 98)	60% (*n* = 1827)
Pregnancy outcome					
CLBR per ovarian stimulation (%)	36% (*n* = 648)	33% (*n* = 233)	31% (*n* = 91)	32% (*n* = 68)	34% (*n* = 1040)
LBR per ET (%)	37% (*n* = 712)	49% (*n* = 239)	45% (*n* = 99)	51% (*n* = 67)	40% (*n* = 1117)

### Prognosis in PGT-M/PGT-SR per ovarian stimulation

The following parameters were considered as potential predictors of CLBR per ovarian stimulation: female age, AMH, BMI, inheritance mode, number of previous unsuccessful PGT cycles, year of treatment and PGT technology (Table 2). The different PGT technologies (PCR, SNP array, array CGH, NGS) used for genetic testing of embryos did not show any association with the expected CLBR per ovarian stimulation. A verification by non-inferiority testing failed to show that SNP array was inferior to PCR technology (*p* = 0.303) (allowing a margin of 5% on the actual estimate) in achieving a live birth per ovarian stimulation. In addition to PGT technology, neither female BMI, the number of previous unsuccessful PGT cycles nor the year of treatment influenced the expected CLBR per ovarian stimulation. On the other hand, the predictors female age, AMH and inheritance mode were found to have a significant influence on the expected CLBR per ovarian stimulation (*p* < 0.001, < 0.001 and < 0.001, respectively). Further post hoc analysis of the parameter ‘inheritance mode’ showed a higher expected CLBR for disorders with an autosomal recessive (AR) inheritance pattern compared to those with an autosomal dominant (AD) or sex-linked (XY) pattern (Table 3).

**Table 2:** Multivariate logistic regression analysis for the cumulative live birth rate per ovarian stimulation. A multivariate logistic regression using female age, AMH, BMI, inheritance mode, number of previous unsuccessful PGT cycles, year of treatment, and PGT technology was performed to analyse potential predictors of the CLBR in women undergoing PGT-M/SR. The intercept represents the PCR technology as well as AR inheritance. Significant p-values are presented in bold. The p-values of BMI, number of previous unsuccessful PGT cycles, and year of treatment were > 0.05 and are therefore not presented. aCGH, array comparative genomic hybridization; NGS, next-generation sequencing; SNPa, single-nucleotide polymorph array; BMI, body mass index; AMH, anti-Mullerian hormone; AR, autosomal recessive; SR, structural rearrangement; XY, sex-linked disorder

	Estimate	std. error	*p*-value
(Intercept)	2300	0.371	** < 0.001**
Technology: aCGH	0.431	0.341	0.206
Technology: NGS	0.354	0.342	0.300
Technology: SNPa	− 0.034	0.101	0.737
Female age	− 0.082	0.010	** < 0.001**
AMH	0.060	0.016	** < 0.001**
Inheritance: AD	− 0.420	0.121	** < 0.001**
Inheritance: SR	− 0.830	0.347	**0.017**
Inheritance: XY	− 0.557	0.176	**0.002**

**Table 3:** Tukey pairwise comparison for inheritance mode. A post hoc analysis, using the Tukey pairwise comparison test, was conducted for the mode of inheritance. Confidence intervals are presented. Confidence intervals not containing zero are presented in bold. AR, autosomal recessive; AD, autosomal dominant; SR, structural rearrangement; XY, sex-linked disorder

	Conf. low	Conf. high
AD–AR	** − 0.724**	** − 0.117**
XY–AR	** − 1.005**	** − 0.108**
SR–AR	− 1.703	0.044
XY–AD	− 0.516	0.244
SR–AD	− 1.251	0.432
SR–XY	− 1.076	0.529

**Fig. 1:**
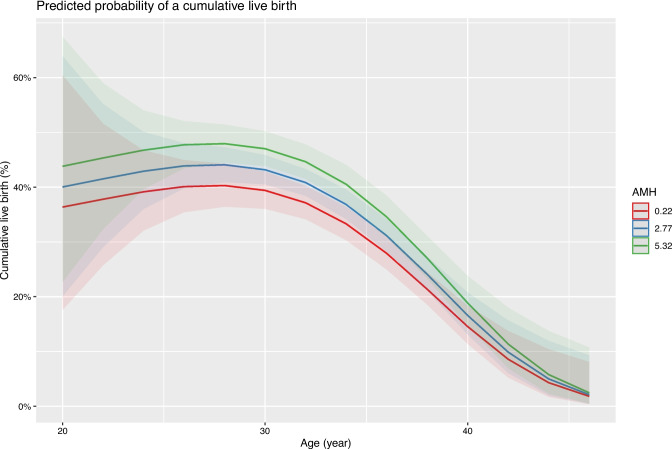
The expected CLBR per ovarian stimulation as a function of female age and AMH. The graph represents the expected CLBR as a function of female age, and for the 25th (in red), the 50th (the median, in blue), and the 75th (in green) percentile of AMH. The shaded areas (in red, blue, and green) represent the 95% confidence interval. An average BMI of 24 kg/m^2^ is assumed in this prediction model

A best-fit model for the expected CLBR per ovarian stimulation was created, with the expected CLBR being dependent on technology, female age which was extended with a polynomial up to the third degree to capture the non-linear relation and AMH. This model suggests that the expected CLBR increases between female ages 20 and 25 years is stable between the ages of 25 and 32 years and shows a rapid decrease after the age of 32 years (Fig. 1).

**Fig. 2:**
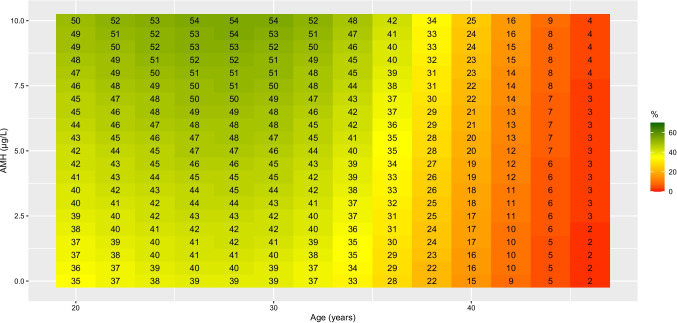
The expected CLBR (in %) per ovarian stimulation based on female age and AMH. The expected CLBR is color-coded depending on prognosis, going from green for good prognosis, to red for very poor prognosis

The absence of a significant difference for expected CLBR per ovarian stimulation between the different PGT technologies allowed us to produce a comprehensive heatmap showing the expected CLBR per ovarian stimulation as a function of female age and AMH values (Fig. 2). In this study, an average woman with AMH = 2.75 µg/L, female age 32.6 years and a BMI of 24.3 kg/m^2^ had an observed CLBR of 41%. The expected CLBR rises with increasing AMH, independent of female age. Furthermore, the trend seen in Fig. 1 was also observed in the heatmap: i.e., an initial rise of expected CLBR up to the age of 28 years, reaching a maximum expected CLBR of 54%, followed by a decrease thereafter. No poor expected CLBRs (≤ 10%) were observed until the female age of 40 years. Poor (≤ 10%) and very poor (≤ 5%) expected CLBRs were observed, for an AMH ≤ 1.25 µg/L, from the female age of 42 and 44 years onwards, respectively. An expected CLBR low enough to be considered futile (≤ 1%) only occurred at a female age of 46 years. The expected CLBR per ovarian stimulation for autosomal dominant disorders, autosomal recessive disorders, sex-linked disorders and chromosomal structural rearrangements are presented in supplementary Figs. 1 to 4, respectively.

### Prognosis in PGT-M/PGT-SR per embryo transfer

**Fig. 3:**
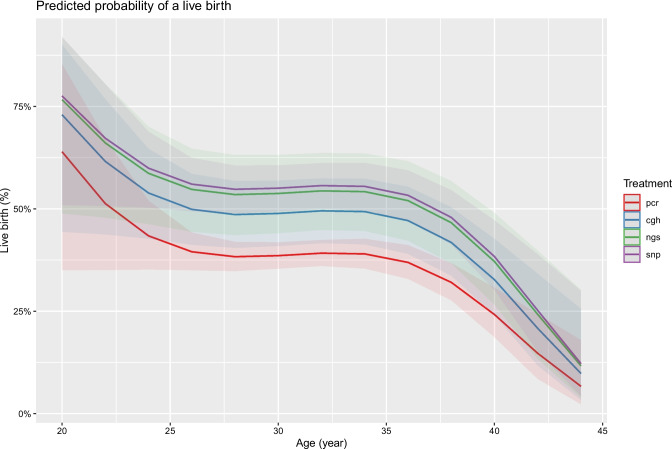
The expected LBR per embryo transfer as a function of female age and the PGT technology used. The graph represents the expected live birth rate per embryo transfer relative to female age and for PCR (in red), array CGH (in blue), NGS (in green), and SNP array (in purple). The shaded areas (in red, blue, green, and purple) represent the 95% confidence interval. For prediction, an average BMI of 24 kg/m^2^ was assumed. LBR, live birth rate; PCR, polymerase chain reaction; aCGH, array comparative genomic hybridization; NGS, next-generation sequencing; SNPa, single-nucleotide polymorphism array

The following parameters were considered as potential predictors of LBR per embryo transfer: female age, BMI, inheritance mode, number of previous unsuccessful PGT cycles, year of treatment and PGT technology. PGT technology and female age (as a polynomial to the third order) were both significant predictors in this model (Table 4). BMI, inheritance mode, number of previous unsuccessful PGT cycles and year of treatment did not show any significant influence on the expected LBR per transfer. Further post hoc analysis of the parameter ‘PGT technology’ showed a lower expected LBR for embryos analyzed using PCR technology (Table 5 and Fig. 3).

**Table 4:** Multivariate logistic regression analysis for the live birth rate per embryo transfer. A multivariate logistic regression using female age, BMI, inheritance mode, number of previous unsuccessful PGT cycles, year of treatment, and PGT technology was performed to analyse the potential predictors of the live birth rate per embryo transfer in women undergoing PGT-M/SR. The intercept represents the PCR technology. Only significant *p*-values are presented. aCGH, array comparative genomic hybridization; NGS, next-generation sequencing; SNPa, single-nucleotide polymorphism array

	Estimate	std. error	*p*-value
(Intercept)	− 0.505	0.058	** < 0.001**
Female age	− 6.156	2.583	**0.017**
Technology: aCGH	0.421	0.167	**0.012**
Technology: NGS	0.612	0.206	**0.003**
Technology: SNPa	0.620	0.122	** < 0.001**

**Table 5:** Tukey pairwise comparison for the LBR per embryo transfer across the different genomic technologies used. A post hoc analysis, using the Tukey pairwise comparison test, was conducted for the PGT technology used. Confidence intervals are presented. Confidence intervals not containing zero are presented in bold. aCGH, array comparative genomic hybridization; PCR, polymerase chain reaction; NGS, next-generation sequencing; SNPa, single-nucleotide polymorph array

	Conf. low	Conf. high
aCGH–PCR	**0.001**	**0.846**
NGS–PCR	**0.089**	**1.136**
SNPa–PCR	**0.311**	**0.929**
NGS–aCGH	− 0.437	0.819
SNPa–aCGH	− 0.282	0.680
SNPa–NGS	− 0.562	0.578

A prognostic model per embryo transfer was created, with the expected LBR being dependent on female age, which was extended with a polynomial up to the third degree to capture the non-linear relation and dependency on PGT technology. This model suggests that the expected LBR per embryo transfer decreases significantly between female age 20 and 25 years is stable between 25 and 35 years, and then decreases again beyond a female age of 35 years. A poor expected LBR per embryo transfer was seen from the age of 44 years onwards for PCR technology, and from the age of 46 years onwards for the other technologies. Very poor or futile expected LBRs were not observed (Fig. 3).

## Discussion

Nowadays, PGT is commonly offered to couples at risk of having a child with a genetic condition. Couples undergoing IVF often have unrealistically high expectations, with a majority expecting a live birth at the first attempt [16, 17]. It should be made clear to couples undergoing PGT varies between IVF centers and has significantly improved over the years, however that the success rate invariably decreases significantly with advanced maternal age and reduced ovarian reserve [6, 18]. Therefore, parameter-based counselling on predicted outcomes may reduce unnecessary treatment as well as the financial, psychological and physical burden associated with PGT trajectories. A previous study from our group identified predictive parameters of the expected CLBR for PGT using a PCR technique and identified women with a poor, very poor or futile prognosis [6]. However, as modern techniques such as SNP array, array CGH or NGS technologies increasingly include comprehensive chromosome screening, there is a need for a more precise prognosis for PGT cycles using parameters such as AMH prior to starting the treatment.

Our data confirmed that the expected ovarian response, estimated based on the AMH level, was a good predictor of the CLBR of PGT treatment [19]. A combination of AMH with female age enables prognostic counselling to provide a more detailed and individualized prognosis prior to PGT. Nevertheless, since AMH is a quantitative measurement, it provides no added value when predicting LBR per embryo transfer. The comprehensive heatmap produced in this study, which visualizes an estimation of the CLBR prior to the PGT treatment, will help identify couples with a futile prognosis as well as attenuate expectations for other couples. During PGT counselling, these couples should be informed of the expected outcome of PGT treatment and alternative reproductive treatment options should be discussed with a health care professional.

Strengths of the present study include the large sample size (3037 PGT cycles), the absence of intervention bias due to a single-center setting, as well as the minimal secular trend in our PGT clinic as confirmed by the absence of year of treatment as a predictor of the (C)LBR.

The parameter ‘female age’ showed a correlation to the third order with the expected CLBR, which reaches a maximum success rate around the age of 28 years, after which it declines. These findings are in line with our observations in an earlier study of the prognosis of conventional IVF and PGT couples [6]. It is well known that women older than 36 years and younger than 25 years carry a higher number of aneuploid oocytes relative to women between these ages and therefore have a lower rate of live births [18, 20]. The parameter ‘female age’ also showed a higher expected LBR for younger women compared to women of advanced maternal age, even when transferring a euploid embryo after SNP array, array CGH or NGS. This age-related decline in expected LBR in the case of euploid embryo transfer has been described earlier [21].

In our study population, no effect of BMI on the expected CLBR could be observed, even though some studies show that a very low or high BMI may negatively influence the expected CLBR [22, 23]. Furthermore, BMI had no evident impact on the expected LBR per embryo transfer, as reported in previous studies [24, 25].

The number of embryos suitable for transfer is influenced by the mode of inheritance of the genetic disorder in question. Depending on this inheritance mode, between 25 and 87.5% of embryos will be unsuitable for transfer due to a genetic burden [3]. Additional aneuploidy screening of embryos may further increase the number of unsuitable embryos. In case of an autosomal recessive disorder, couples should be able to use 75% of all well-developed embryos, whereas couples with an autosomal dominant disorder can expect 50% of all embryos to be genetically suitable for transfer. Couples undergoing PGT for X-linked disorders often opt to transfer only those embryos without the genetic variant, as in some cases female offspring may be affected by the disorder due to unfavorable skewing of X-inactivation. This will result in transfer of only 50% of all embryos. As we showed in this study, couples with an autosomal recessive disorder tend to have a higher expected CLBR compared to those with an autosomal dominant disorder or sex-linked disorder. The various types of structural rearrangements, such as reciprocal translocations, Robertsonian translocations, inversions or complex chromosomal rearrangements, can lead to distinct meiotic segregation patterns during gametogenesis and as a result lead to varying proportions of balanced embryos suitable for transfer. Unfortunately, the size of our current cohort did not allow us to perform sub-analyses for each type of structural rearrangement. To gain insights into these specific success rates, we require larger databases to provide more comprehensive information. Furthermore, some individuals or couples, carriers of chromosomal structural rearrangements, opt to transfer embryos with a normal chromosomal constitution only, while others transfer both embryos that are normal and balanced for the translocation. Again, our current cohort size limits sub-analyses. Larger datasets are essential to investigate outcomes in these different cohorts. As decisions regarding the embryo transfer policy in each couple have already been taken beforehand, the mode of inheritance will not influence the expected LBR at the moment of embryo transfer.

We observed that the PGT technology used to test embryos did not influence the expected CLBR. This observation implies that the choice between cleavage stage biopsy followed by PCR, and trophectoderm biopsy followed by SNP array for PGT-M will not affect the outcome per ovarian stimulation, neither will the choice between SNP array, array CGH or NGS for PGT-SR. Our cohort’s findings diverged from previous studies, as we observed no significant impact on expected CLBR when comparing cleavage stage biopsy to trophectoderm biopsy [26]. In an experienced lab, it appears that the embryonic stage for biopsy does not influence the expected CLBR. Per embryo transfer, however, embryos analyzed by PCR have a lower chance of resulting in a live birth compared to embryos analyzed using genome-wide technologies, as the latter embryos are only transferred when considered to be euploid. Therefore, caution is warranted when interpreting the LBR from euploid embryos as opposed to those that may not be euploid.

While the model presented in this study serves as a predictive tool based on specific clinical parameters, a limitation remains that scores for any individual are influenced by factors not represented in our model, uncertainty in the prediction of theses individual scores appears unavoidable. For instance, we did not include variables such as endometrium preparation protocol, endometrium thickness and hormonal milieu in our analysis for estimating the CLBR. In addition, this prognostic model does not apply to couples with a genetic variant that affects ovarian response (e.g. *FMR1* premutations or rearrangements involving the sex chromosomes), or to couples undergoing PGT-A. PGT centers should develop center-specific prognostic heatmaps as reproductive outcome may vary according to different patient populations and laboratory expertise.

## Conclusions

We developed a heatmap to visualize the expected CLBR for PGT-M and PGT-SR couples. In a PGT trajectory, couples should be clearly informed about the prognosis, especially in the event of an unfavorable combination of female age and AMH. In such cases, couples should be counselled regarding other reproductive choices.

### Supplementary information

Below is the link to the electronic supplementary material.

Supplementary file1 Supplementary figure 1 The expected CLBR (in %) for autosomal dominant disorders per ovarian stimulation based on female age and AMH. A color code is given to the expected CLBR depending on the prognosis, going from green for good prognosis, to red for very poor prognosis. (JPG 2656 KB)

Supplementary file2 Supplementary figure 2 The expected CLBR (in %) for autosomal recessive disorders per ovarian stimulation based on female age and AMH. A color code is given to the expected CLBR depending on the prognosis, going from green for good prognosis, to red for very poor prognosis. (JPG 2598 KB)

Supplementary file3 Supplementary figure 3 The expected CLBR (in %) for sex linked disorders per ovarian stimulation based on female age and AMH. A color code is given to the expected CLBR depending on the prognosis, going from green for good prognosis, to red for very poor prognosis. (JPG 2669 KB)

Supplementary file4 Supplementary figure 4 The expected CLBR (in %) for chromosomal structural rearrangements per ovarian stimulation based on female age and AMH. A colour code is given to the expected CLBR depending on the prognosis, going from green for good prognosis, to red for very poor prognosis. (JPG 2648 KB)

## Data Availability

All data underlying this study will be made available on request.
