# Supplementary figures and images for: A heatmap for expected cumulative live birth rate in preimplantation genetic testing for monogenic disorders and chromosomal structural rearrangements
Source: J Assist Reprod Genet. 2024 May 16;41(7):1907–15. doi: 10.1007/s10815-024-03141-6 (PMC11263274; doi:10.1007/s10815-024-03141-6)

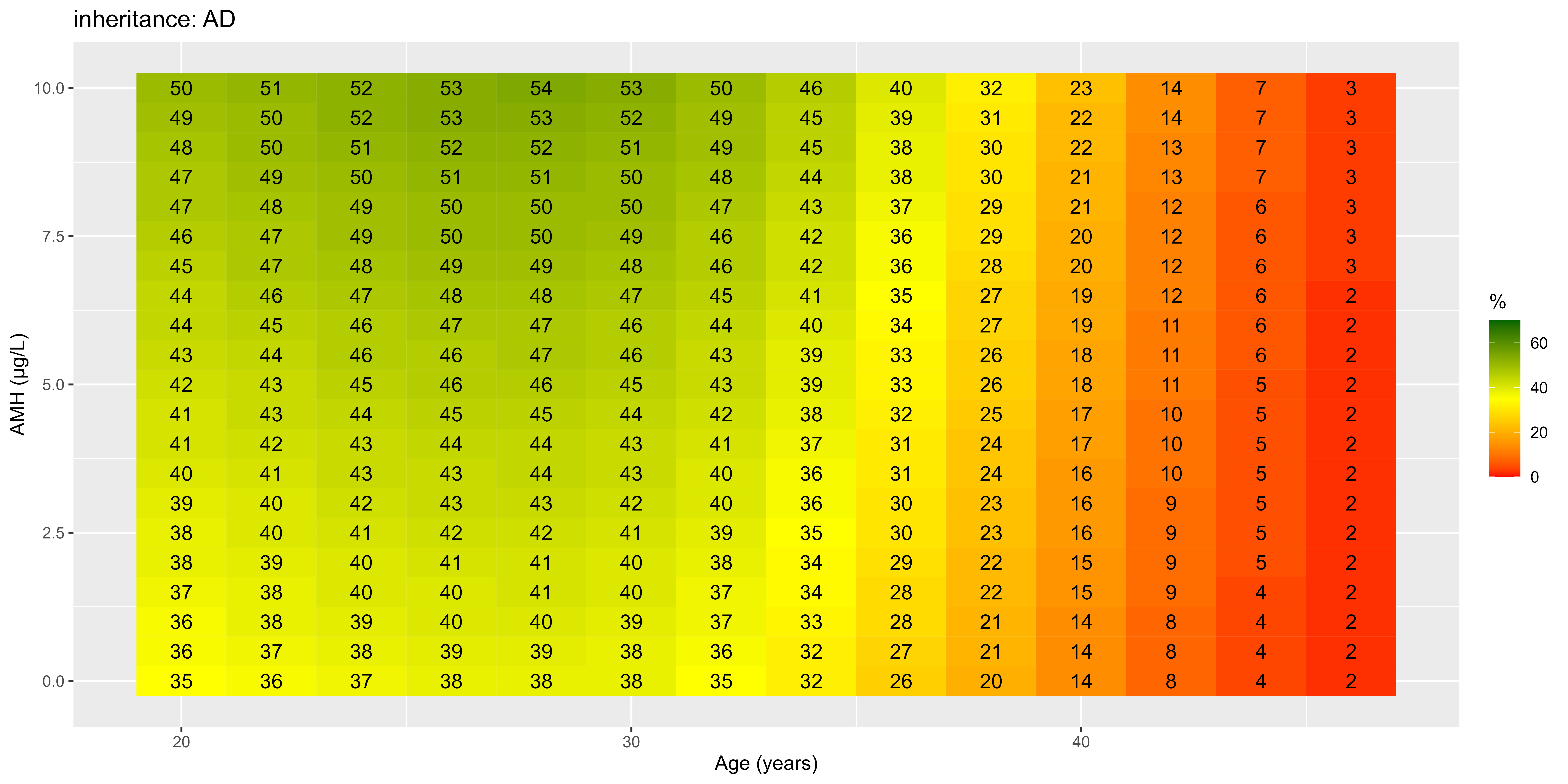

Supplement: Supplementary file 1 — Supplementary file1 Supplementary figure 1 The expected CLBR (in %) for autosomal dominant disorders per ovarian stimulation based on female age and AMH. A color code is given to the expected CLBR depending on the prognosis, going from green for good prognosis, to red for very poor prognosis. (JPG 2656 KB) [file 10815_2024_3141_MOESM1_ESM.jpg]

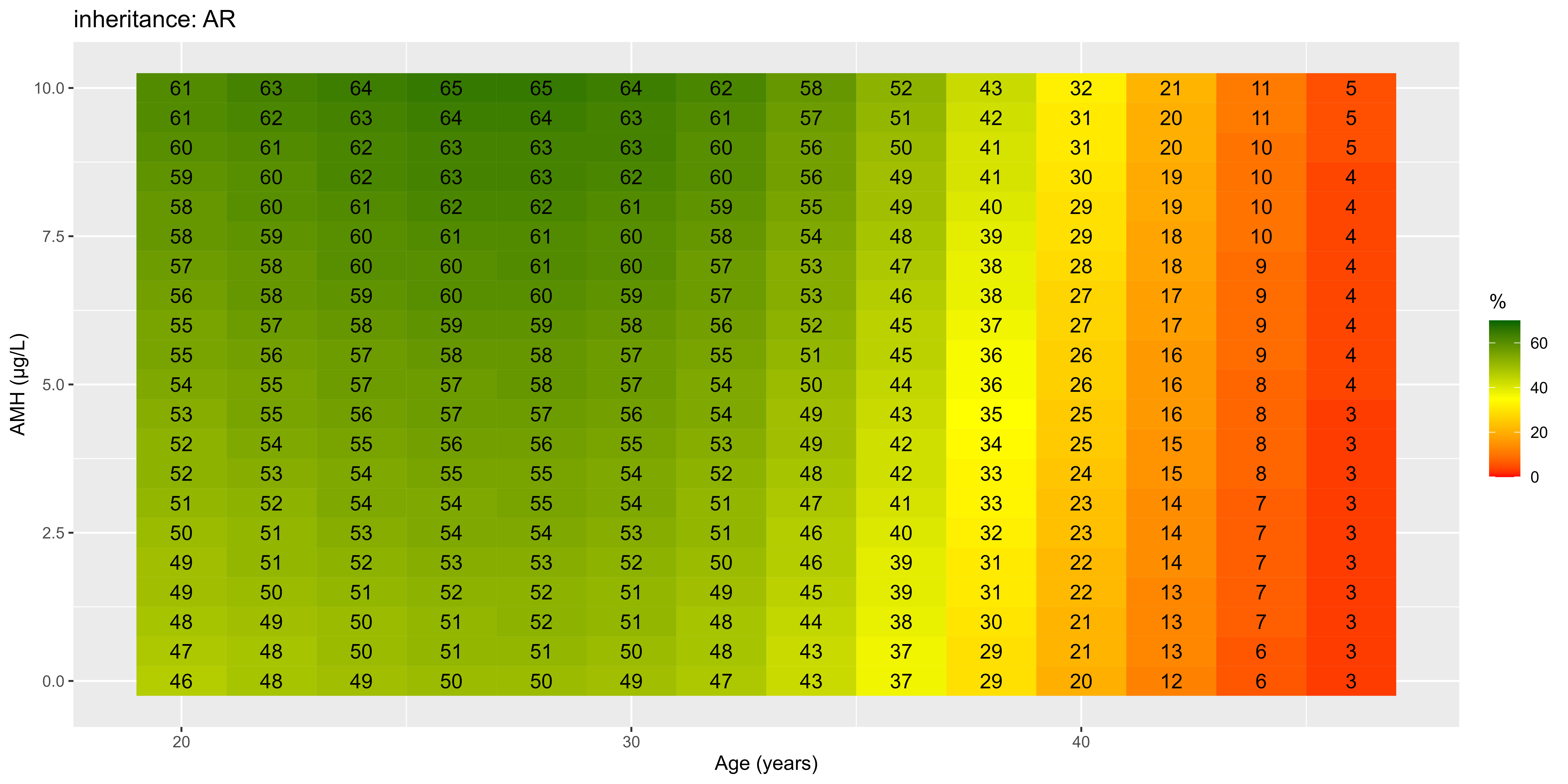

Supplement: Supplementary file 2 — Supplementary file2 Supplementary figure 2 The expected CLBR (in %) for autosomal recessive disorders per ovarian stimulation based on female age and AMH. A color code is given to the expected CLBR depending on the prognosis, going from green for good prognosis, to red for very poor prognosis. (JPG 2598 KB) [file 10815_2024_3141_MOESM2_ESM.jpg]

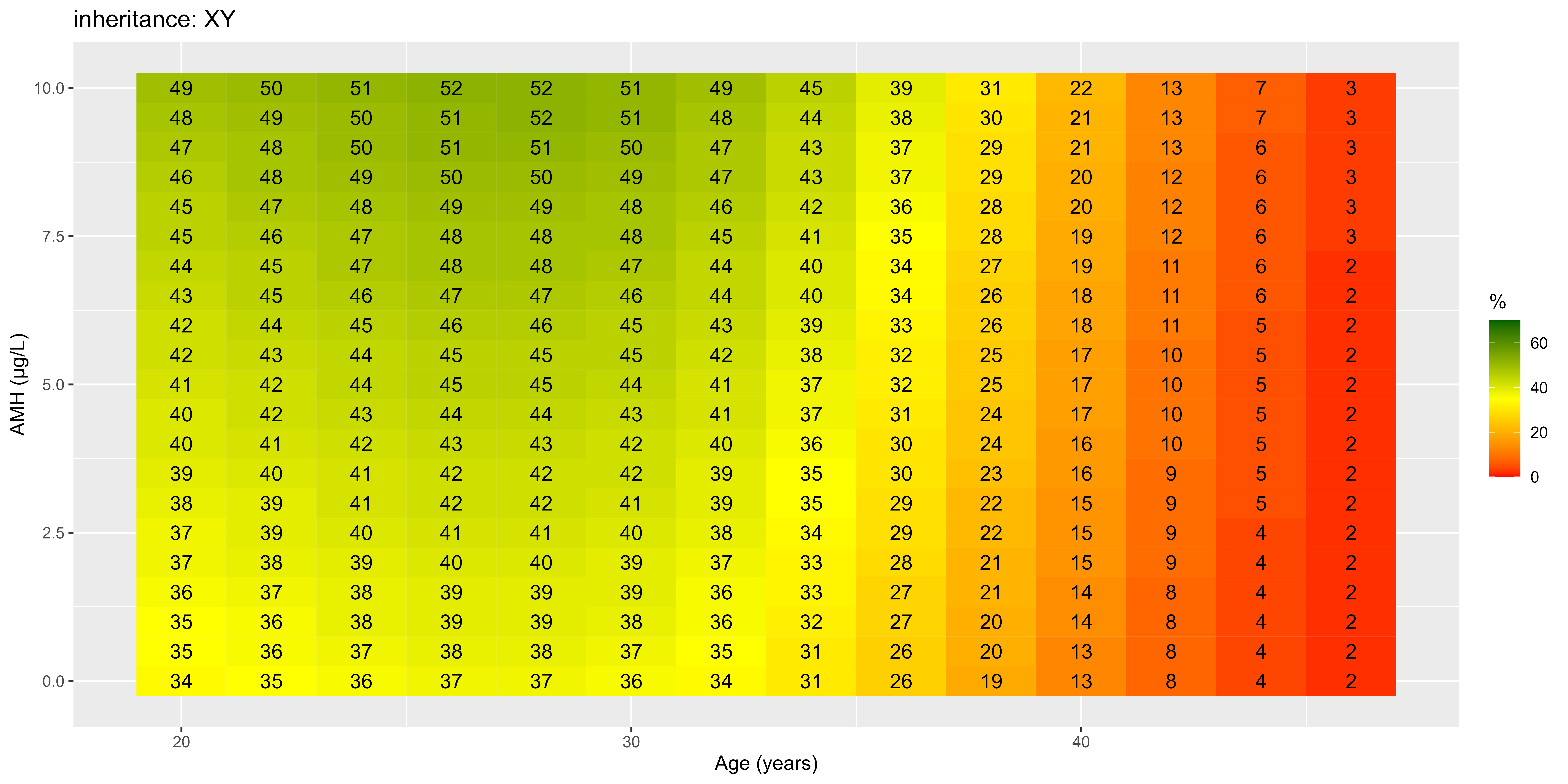

Supplement: Supplementary file 3 — Supplementary file3 Supplementary figure 3 The expected CLBR (in %) for sex linked disorders per ovarian stimulation based on female age and AMH. A color code is given to the expected CLBR depending on the prognosis, going from green for good prognosis, to red for very poor prognosis. (JPG 2669 KB) [file 10815_2024_3141_MOESM3_ESM.jpg]

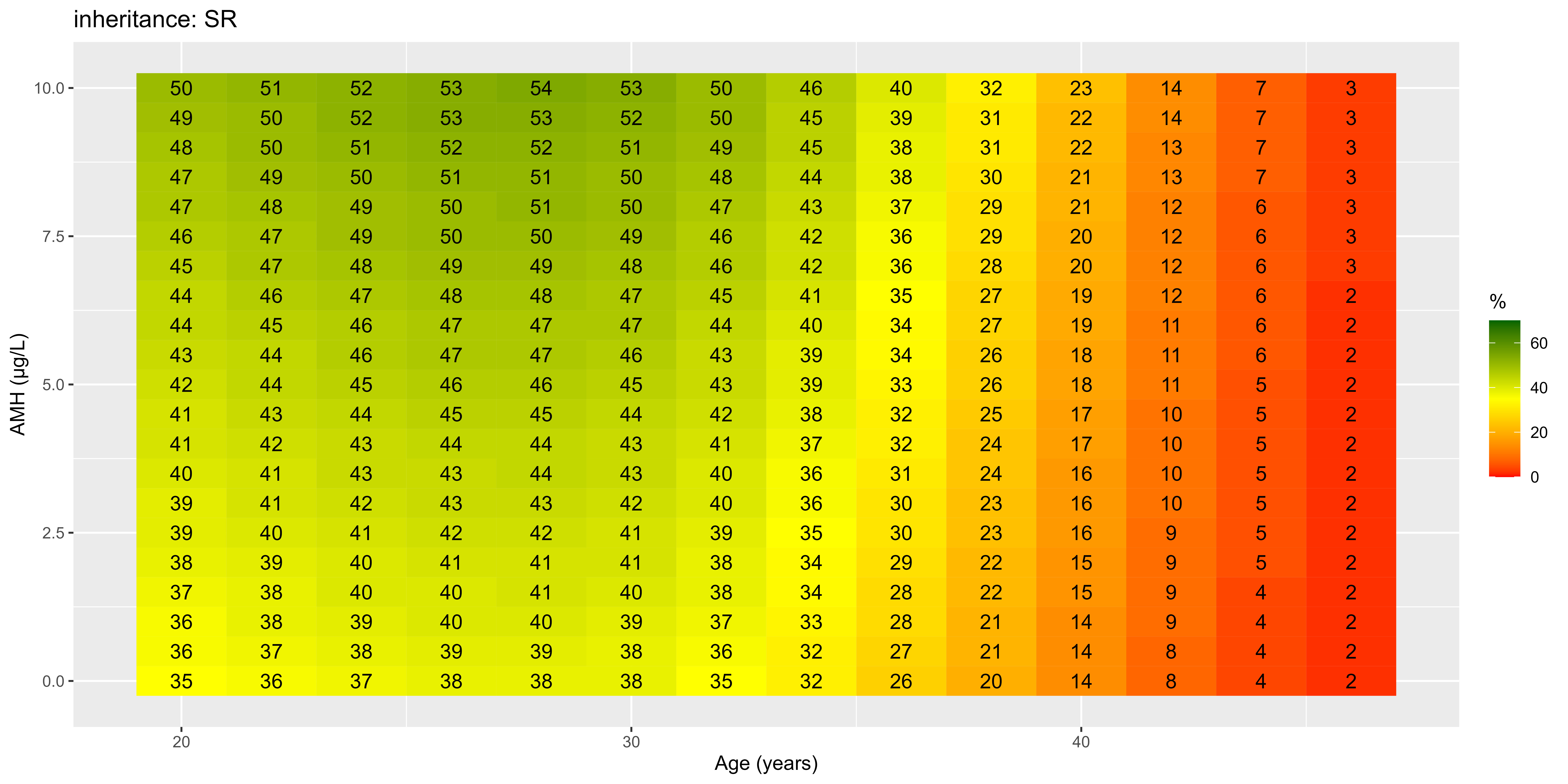

Supplement: Supplementary file 4 — Supplementary file4 Supplementary figure 4 The expected CLBR (in %) for chromosomal structural rearrangements per ovarian stimulation based on female age and AMH. A colour code is given to the expected CLBR depending on the prognosis, going from green for good prognosis, to red for very poor prognosis. (JPG 2648 KB) [file 10815_2024_3141_MOESM4_ESM.jpg]
